# Linear IgA bullous dermatosis secondary to drugs: a real-world pharmacovigilance study of the FDA adverse event reporting system

**DOI:** 10.3389/fmed.2025.1521697

**Published:** 2025-01-23

**Authors:** Yixuan Yang, Hanzhang Xie, Shuhan Liu, Ying Jia, Bingnan Cui, Zhanshuo Xiao

**Affiliations:** ^1^Department of Dermatology, Guang’anmen Hospital, China Academy of Chinese Medical Sciences, Beijing, China; ^2^Beijing University of Chinese Medicine, Beijing, China

**Keywords:** linear IgA bullous dermatosis, drug-induced linear IgA bullous dermatosis, adverse event, FAERS, pharmacovigilance study

## Abstract

**Background:**

Linear IgA bullous dermatosis (LABD) is a rare autoimmune blistering disease. The induction of LABD by medications is a critical issue, with previous studies highlighting the link between specific drugs and the onset of LABD. This study aims to assess the reported associations between LABD and numerous available medications using the FDA adverse event reporting system (FAERS).

**Methods:**

The study encompassed FAERS reports spanning the years 2004–2024. Medical Dictionary for Regulatory Activities (MedDRA) was used to identify cases of LABD. The Reporting Odds Ratio, Proportional Reporting Ratio, Bayesian Confidence Propagation Neural Network, and Empirical Bayes Geometric Mean were calculated to assess the reported associations between available drugs and LABD. A significant statistical association was considered when a drug signal met the criteria of all four algorithms.

**Results:**

In the FAERS database analysis, we identified 1,394 adverse event (AE) reports associated with LABD. The gender distribution of reports was relatively balanced, with the highest proportion in the 66–85 age group. The United States had the highest number of reports. Vancomycin and Amoxicillin were the most frequently reported drugs, with 559 and 58 reports, respectively. Through disproportionality analysis, we identified 34 drugs significantly associated with AEs of LABD, including antibiotics, antifungal medications, analgesics, nonsteroidal anti-inflammatory drugs, cardiovascular medications, and calcium channel blockers, among which the antibiotic Vancomycin showed the highest association. These results emphasize the need for further clinical attention to the safety of specific medications.

**Conclusion:**

This is the first real-world study using the FAERS database to investigate drug-induced LABD. LABD is closely associated with antibiotic medications. Close monitoring of patients is required when these medications are used clinically to promptly detect and manage potential AEs such as LABD.

## 1 Introduction

Linear IgA Bullous Dermatosis (LABD) is a rare immune-mediated bullous disease affecting both pediatric and adult populations (1). Pediatric patients frequently exhibit annular or polycyclic plaques, papules, and peripheral vesicles, known as the pearl string sign, predominantly on the face, genitals, and extremities (2). In adults, the lesions are primarily localized to the trunk, head, and extremities (3). Mucosal involvement is observed in both pediatric and adult cases, although the pearl string sign is less frequently observed in adults (2). Subjective symptoms range from mild itching to severe stinging (3, 4). Diagnosis relies on direct immunofluorescence testing of skin biopsy, showing linear immunoglobulin A (IgA) deposition along the basement membrane zone (5–7), occasionally accompanied by deposits of IgG, IgM, and C3 (5). The incidence of LABD is approximately 0.2 to 2.3 cases per million people per year (8–11).

LABD can be categorized into idiopathic and drug-induced forms. Idiopathic LABD typically occurs spontaneously, but it may also be associated with inflammatory bowel disease, malignancies, infections, and other autoimmune disorders (12). Although the majority of cases are idiopathic, various medications are known to induce LABD, with vancomycin being the most common precipitating drug (13). In addition, nonsteroidal anti-inflammatory drugs (NSAIDs), penicillins, cephalosporins, diuretics, and anticonvulsants are also common triggers for LABD (14). The clinical manifestations of both idiopathic and drug-induced LABD are diverse, with characteristics that can resemble those of pityriasis lichenoides et varioliformis acuta, bullous pemphigoid, pemphigus vulgaris, erythema multiforme, and toxic epidermal necrolysis (TEN) (5, 7, 15). Skin and mucosal symptoms in patients with drug-induced LABD are essentially similar to those with idiopathic LABD (16, 17), including vesicular rash, erythematous patches, target lesions, and string of pearls sign (17, 18). However, drug-induced LABD tends to be more severe, extensive, and atypical. The frequency of positive Nikolsky sign and large erosions is higher in drug-induced LABD, sometimes clinically resembling TEN (13). Several reports have indicated that drug-induced LABD may clinically overlap with TEN or Stevens-Johnson syndrome/toxic epidermal necrolysis (SJS/TEN) (15, 19–22), thus drug-induced LABD may pose a potential life-threatening risk. Therefore, early assessment and detection of signals for drug adverse events (AEs) are crucial for reducing the risk of drug-induced LABD.

To our knowledge, no studies have specifically utilized the FDA adverse event reporting system (FAERS) database to explore drug-induced LABD. The aim of this study is to assess the reported associations between drugs and LABD using the FAERS database.

## 2 Methods

### 2.1 Data source

This retrospective pharmacovigilance study utilizes the FAERS database, a global resource for post-marketing drug safety monitoring and signal detection. The database comprises reports submitted voluntarily by healthcare providers, as well as mandatory submissions from pharmaceutical companies. Information on drugs, including their names, active ingredients, routes of administration, and roles in adverse events (AEs), along with codes for different drug interactions such as primary suspect (PS), secondary suspect, interacting, and concomitant medications, is accessible within the FAERS. Each report identifies a primary suspect drug, enumerates one or more adverse drug reactions (ADRs), and may detail additional medications ingested by the patient.

### 2.2 Study design

This retrospective pharmacovigilance study encompassed FAERS data from January 2004 to June 2024. To account for multiple submissions with updated information, duplicate reports were identified and excluded based on case numbers, with only the most recent version retained for analysis. A case–control analysis was performed using FAERS to investigate the association between drug exposure and LABD reports. In this analysis, ‘cases’ corresponded to reports of AEs of interest, while ‘controls’ comprised all other AE reports not related to the AE under scrutiny. Classification of cases and controls was based on exposure or non-exposure to the drug in question. The reporting odds ratio (ROR) and its 95% confidence interval (CI) were calculated as a measure of association. The ROR specifically indicates whether an AE is disproportionately reported in relation to all other AEs associated with a particular exposure, thus reflecting the reporting odds of the AE of interest between those exposed and those not exposed to the drug. Additionally, the proportional reporting ratio (PRR), Bayesian Confidence Propagation Neural Network (BCPNN), and Empirical Bayes Geometric Mean (EBGM) were employed to detect drug signals.

### 2.3 Data exposure and adverse drug reaction definition

This study used the preferred term ‘LINEAR IGA DISEASE’ from the Medical Dictionary for Regulatory Activities (MedDRA) to identify AEs of LABD in the REACTION (REAC) files. Given that reporters can assign various roles to the drugs in question, the assessment of drug exposure focused solely on those designated with the ‘PS’ role code. DrugBank was used to standardize different drug names in the ‘drugs’ table, such as brand names, generic names, synonyms, or abbreviations. All drugs ultimately appeared in the standardized generic name format.

### 2.4 Statistical analysis

Disproportionate analysis is extensively applied for identifying signals of ADRs (23). In this study, we employed four analytical approaches, conducting statistical analyses based on the construction of a 2 × 2 contingency table (24). The methodologies encompassed both frequency-based metrics, namely the ROR and the PRR, and Bayesian methodologies, including the BCPNN and the EBGM. The Bayesian methods, while computationally more intensive, offer a significant advantage over the frequency-based methods by mitigating the risk of false positives associated with sparse AE reporting (25). The synergistic application of these four algorithms enhances the robustness and reliability of the findings. All data processing and statistical analyses were performed utilizing R software, version 4.4.1.

## 3 Results

### 3.1 Case characteristics

**Figure 1:**
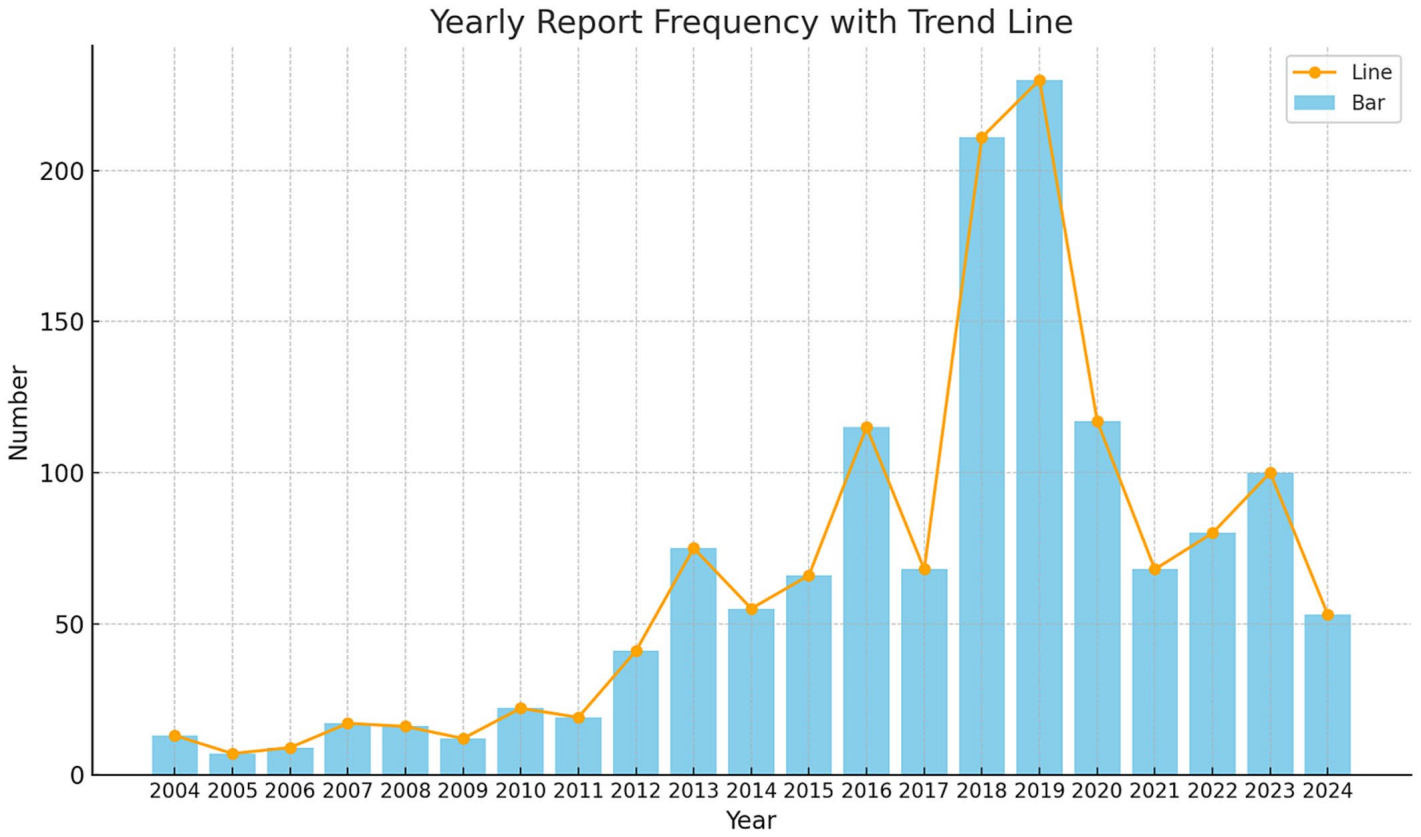
Annual distribution of reports associated with linear IgA bullous dermatosis collected from the FAERS database between January 2004 and June 2024.

Between the first quarter of 2004 and the second quarter of 2024, our study extracted a total of 21,433,114 AE reports from the FAERS database. After removing duplicates, we obtained 18,182,912 distinct AE reports. Ultimately, 1,394 reports related to LABD were identified. Figure 1 illustrates the annual submission of AE reports, showing a peak in 2019, potentially associated with the administration of COVID-19 vaccines or related medications (26–29). In the analyzed reports, there was a slight male predominance (44.4%) compared to females (43.2%). In terms of weight, patients weighing 50–100 kg accounted for 4.9%. Regarding age, the majority of patients were in the 66–85 age range (37.0%), followed by those aged 18–65 (34.5%). The majority of reports were submitted by Physicians (38.5%) and other healthcare professionals (32.6%). Geographically, the United States accounted for the highest proportion of reports (26.0%), followed by France (21.5%), Japan (7.7%), Great Britain (5.1%), and Spain (3.6%). Regarding outcomes, the majority of reports indicated other serious outcomes (56.0%), followed by hospitalization (33.0%) and death (5.7%). The most frequently reported indications were Pneumonia (4.3%), Infection (3.9%), Hypertension (2.9%), Endocarditis (1.9%), and Staphylococcal infection (1.9%). Table 1 presents detailed clinical characteristics of the reports associated with LABD.

**Table 1:** Clinical characteristics of linear IgA bullous dermatosis - related reports collected from the FAERS database between January 2004 and June 2024.

Characteristics	Case number	Case proportion
Number of events	1,394	
Sex		
F	602	43.2%
M	619	44.4%
Missing	173	12.4%
Weight (kg)		
<50	16	1.1%
>100	25	1.8%
50 ~ 100	68	4.9%
Missing	1,285	92.2%
Age (year)		
<18	102	7.3%
>85	94	6.7%
18–65	481	34.5%
66–85	516	37.0%
Missing	201	14.4%
Reported person		
Consumer	25	1.8%
Health ProfeLABDional	191	13.7%
Pharmacist	60	4.3%
Other health-profeLABDional	455	32.6%
Physician	537	38.5%
Missing	126	9.0%
Reported countries (top five)		
United States	363	26.0%
France	300	21.5%
Japan	107	7.7%
Great Britain	71	5.1%
España	50	3.6%
Outcome		
Death	79	5.7%
Life-threatening	38	2.7%
Hospitalization	460	33.0%
Disability	9	0.6%
Other Serious	780	56.0%
Missing	22	1.6%
Indications (top five)		
Pneumonia	60	4.30%
Infection	54	3.9%
Hypertension	41	2.9%
Endocarditis	26	1.9%
Staphylococcal infection	26	1.9%

### 3.2 Medications used for LABD

Among the 1,394 AE reports associated with LABD identified in this study, a total of 353 unique drug names were listed as “PS.” After merging different names, including brand and generic names, we arrived at 175 distinct medications. Vancomycin topped the list with 559 reports, followed by Amoxicillin (*n* = 58), Amlodipine (*n* = 56), Piperacillin and Tazobactam (*n* = 40), and Diclofenac (*n* = 33) (Table 2). We performed a disproportionality analysis on the 175 medications with more than five reports and initially identified 37 drugs that met the criteria of the ROR algorithm. To more accurately elucidate the relationship between medications and AEs of LABD, we further analyzed drugs that satisfied all four disproportionality analysis methods (Table 3). Among the 34 drugs with significant associations, we found a variety of drug classes, including antibiotics, antifungal medications, analgesics, NSAIDs, cardiovascular medications, and calcium channel blockers. Notably, Ampicillin-Sulbactam and Ketoprofen had elevated RORs of 137.77 and 109.21, respectively, indicating a strong link to AEs of LABD. Vancomycin demonstrated the most significant signal with an ROR of 692.12 (95% CI 621.74–770.46), indicating a potentially strong association with AEs. Piperacillin-Tazobactam had an ROR of 73.62, emphasizing the need for safety monitoring. Although Gabapentin (*n* = 12, ROR 2.65) and Cyclosporine (*n* = 19 ROR 2.66) did not meet all four disproportionality analyses, their relatively high report numbers suggest that they warrant further clinical vigilance. While the four disproportionality analyses provide a more stable and reliable association, drugs with a high number of AE reports in the real world that meet even a single statistical method still merit further clinical attention and vigilance.

**Table 2:** Drugs linked to linear IgA bullous dermatosis with more than five reports (highlighting signals meeting the ROR method in bold).

DRUG	Case numbers	ROR
**Vancomycin**	559	**692.12**
**Amoxicillin**	58	**23**
**Amlodipine**	56	**11.49**
**Piperacillin and Tazobactam**	40	**73.62**
**Diclofenac**	33	**5.32**
**Amoxicillin and Clavulanic**	30	**42**
**Ciprofloxacin**	28	**6.51**
**Atorvastatin**	27	**6.7**
**Ibuprofen**	22	**4.25**
**Sulfamethoxazole and Trimethoprim**	21	**25.8**
**Metronidazole**	20	**16.67**
**Meloxicam**	17	**36.38**
**Levofloxacin**	15	**6.02**
**Cefuroxime**	14	**27.53**
**Omeprazole**	14	**4.9**
**Azithromycin**	13	**7.89**
**Furosemide**	13	**6.63**
**Gabapentin**	12	**2.65**
**Simvastatin**	12	**4.67**
**Ceftriaxone**	11	**17.72**
**Meropenem**	11	**39.67**
**Phenytoin**	11	**11.77**
**Allopurinol**	10	**12.63**
**Ketoprofen**	10	**109.21**
**Rifampin**	10	**16.56**
**Cyclosporine**	9	**2.66**
**Fluconazole**	9	**13.07**
Metformin	9	1.32
**Terbinafine**	9	**17.12**
**Ampicillin and Sulbactam**	8	**137.77**
**Bisoprolol**	8	**6.92**
Esomeprazole	8	1.24
**Gemcitabine**	8	**4.95**
Infliximab	7	0.41
**Imipenem and Cilastatin**	7	**27.54**
**Donepezil**	6	**9**
**Paracetamol**	6	**4.76**
**Sitagliptin**	6	**3.43**
**Verapamil**	6	**11.44**
**Captopril**	5	**99.12**
Nivolumab	5	1.1
Rosuvastatin	5	1.1
Ustekinumab	5	1.23

**Table 3:** Drugs significantly linked to linear IgA bullous dermatosis with more than five reports (meeting the criteria of all four disproportionality analysis methods).

DRUG	Case numbers	ROR (95%Cl)	PRR (χ2)	EBGM (EBGM05)	IC (IC025)
Vancomycin	559	692.12 (621.74–770.46)	684.68 (229575.88)	412.28 (376.9)	8.69 (7.02)
Ampicillin and Sulbactam	8	137.77 (68.68–276.38)	137.28 (1076.19)	136.51 (76.24)	7.09 (5.42)
Ketoprofen	10	109.21 (58.58–203.61)	108.91 (1061.58)	108.14 (64.21)	6.76 (5.09)
Captopril	5	99.12 (41.15–238.79)	98.87 (482.68)	98.52 (47.21)	6.62 (4.95)
Piperacillin and Tazobactam	40	73.62 (53.75–100.85)	73.49 (2778.57)	71.42 (54.89)	6.16 (4.49)
Amoxicillin and Clavulanic	30	42 (29.24–60.31)	41.95 (1173.67)	41.08 (30.34)	5.36 (3.69)
Meropenem	11	39.67 (21.91–71.82)	39.63 (410.99)	39.33 (23.93)	5.3 (3.63)
Meloxicam	17	36.38 (22.55–58.71)	36.35 (577.35)	35.92 (24.07)	5.17 (3.5)
Imipenemand and Cilastatin	7	27.54 (13.1–57.89)	27.52 (177.99)	27.39 (14.71)	4.78 (3.11)
Cefuroxime	14	27.53 (16.26–46.62)	27.51 (354.14)	27.25 (17.54)	4.77 (3.1)
Sulfamethoxazole and Trimethoprim	21	25.8 (16.76–39.7)	25.78 (492.79)	25.41 (17.72)	4.67 (3)
Amoxicillin	58	23 (17.68–29.91)	22.98 (1169.21)	22.08 (17.72)	4.46 (2.8)
Ceftriaxone	11	17.72 (9.79–32.08)	17.71 (172.1)	17.58 (10.7)	4.14 (2.47)
Terbinafine	9	17.12 (8.89–32.97)	17.11 (135.64)	17.01 (9.83)	4.09 (2.42)
Metronidazole	20	16.67 (10.72–25.92)	16.66 (290.26)	16.44 (11.36)	4.04 (2.37)
Rifampin	10	16.56 (8.89–30.85)	16.55 (145.09)	16.44 (9.77)	4.04 (2.37)
Fluconazole	9	13.07 (6.79–25.18)	13.07 (99.67)	12.99 (7.51)	3.7 (2.03)
Allopurinol	10	12.63 (6.78–23.53)	12.63 (106.28)	12.54 (7.45)	3.65 (1.98)
Phenytoin	11	11.77 (6.5–21.31)	11.77 (107.56)	11.69 (7.11)	3.55 (1.88)
Amlodipine	56	11.49 (8.79–15.01)	11.48 (514.61)	11.07 (8.85)	3.47 (1.8)
Verapamil	6	11.44 (5.13–25.52)	11.44 (56.91)	11.39 (5.82)	3.51 (1.84)
Donepezil	6	9 (4.04–20.08)	9 (42.49)	8.97 (4.58)	3.16 (1.5)
Azithromycin	13	7.89 (4.57–13.62)	7.89 (77.46)	7.82 (4.95)	2.97 (1.3)
Bisoprolol	8	6.92 (3.45–13.87)	6.92 (40.29)	6.89 (3.85)	2.78 (1.12)
Atorvastatin	27	6.7 (4.58–9.81)	6.7 (128.49)	6.59 (4.79)	2.72 (1.05)
Furosemide	13	6.63 (3.84–11.45)	6.63 (61.58)	6.58 (4.17)	2.72 (1.05)
Ciprofloxacin	28	6.51 (4.48–9.47)	6.51 (128.04)	6.4 (4.68)	2.68 (1.01)
Levofloxacin	15	6.02 (3.62–10.02)	6.02 (62.15)	5.97 (3.9)	2.58 (0.91)
Diclofenac	33	5.32 (3.77–7.52)	5.32 (113.15)	5.22 (3.91)	2.38 (0.72)
Gemcitabine	8	4.95 (2.47–9.93)	4.95 (25.1)	4.93 (2.76)	2.3 (0.63)
Omeprazole	14	4.9 (2.9–8.3)	4.9 (43.06)	4.86 (3.13)	2.28 (0.61)
Paracetamol	6	4.76 (2.14–10.62)	4.76 (17.76)	4.75 (2.43)	2.25 (0.58)
Simvastatin	12	4.67 (2.65–8.25)	4.67 (34.37)	4.64 (2.89)	2.22 (0.55)
Ibuprofen	22	4.25 (2.79–6.48)	4.25 (53.8)	4.2 (2.95)	2.07 (0.4)

## 4 Discussion

LABD is an autoimmune blistering disease with linear IgA deposits at the basement membrane. It can be categorized into idiopathic and drug-induced forms. In idiopathic LABD, LAD285, BP180, and BP230 have been identified as the primary target antigens, with the NC16A domain of BP180 being the main target for antibodies (30). In drug-induced LABD, antibodies against LAD285 and BP180 are present (17). Drugs may provoke autoimmune responses by mimicking epitopes, changing their structure, or revealing hidden antigens (31–33).

The pathogenesis of drug-induced LABD remains unclear. Drug-specific T cells and their cytokines, such as interleukins IL-4, IL-5, IL-6, IL-10, and transforming growth factor *β*, may augment IgA synthesis (34). Cytotoxic CD8^+^ T lymphocytes are thought to be central in recognizing self-antigens in drug-induced LABD (35). An increase in activated CD8^+^ T cells in the peripheral blood correlates with onset of LABD (35). IL-5, with elevated levels and local skin expression, is significant in drug-specific T cell responses, promoting IgA class switching and eosinophil activation, which are pivotal in tissue damage and blister formation. Elevated IFN-*γ* levels suggest the activation of cytotoxic T cells, potentially worsening tissue damage and triggering autoimmune processes that manifest as LABD (36). Cytotoxic mechanisms are common in drug-induced skin diseases (37, 38).

Clinical characteristic data from LABD reports highlight several key points. The gender distribution slightly favors males over females, although the difference is not significant. The significant lack of weight data limits our ability to analyze the potential link between obesity and LABD. The age distribution indicates that LABD can affect patients across all age groups but is most prevalent in adults, particularly in the 66–85 age range. This suggests a need for increased caution when treating elderly patients, considering the higher likelihood of comorbidities and polypharmacy in this age group. The diversity of reporting sources reflects a global awareness and willingness to report LABD, with physicians reporting the highest number of cases, underscoring their pivotal role in identifying and reporting adverse drug reactions. Geographically, the United States, France, Japan, Great Britain, and Spain report the most cases, which may correlate with pharmaceutical market distribution, regulatory capabilities, and demographic factors in these countries. High hospitalization rates underscore the severity of LABD, indicating the need for vigilant monitoring of LABD signs during drug use. Although rare, fatal cases remind us of the potential lethal risks associated with LABD. More than half of the patients (56.0%) reported severe clinical outcomes, further emphasizing the importance of early identification and treatment of LABD. Lastly, indications are primarily for the treatment of inflammatory diseases and infections, suggesting that certain medications used in these conditions may be associated with the occurrence of LABD. In particular, antibiotics such as Vancomycin and NSAIDs like Ketoprofen are significantly associated with LABD, and further research is needed to explore their potential mechanisms.

Drug-induced LABD is typically characterized by spontaneous resolution following drug withdrawal (39–41). The six drugs most commonly implicated in LABD in the FAERS database are Vancomycin (*n* = 559), followed by Amoxicillin (*n* = 58), Amlodipine (*n* = 56), Piperacillin and Tazobactam (*n* = 40), Diclofenac (*n* = 33), and Amoxicillin and Clavulanic (*n* = 30). The high ROR and PRR values for these drugs indicate a statistically significant association with LABD. For instance, Vancomycin, Ampicillin and Sulbactam, and Ketoprofen have ROR values of 692.12, 137.77, and 109.21, respectively, suggesting an increased risk of LABD in specific patient populations. Furthermore, the EBGM and Information Component (IC) values confirm the consistency and biological plausibility of these associations. Vancomycin, for instance, has an EBGM value of 412.28 and an IC value of 8.69, reinforcing its strong link with LABD. The findings are not surprising, as our research also indicates that antibiotics are the most representative drug class in drug-induced LABD. Vancomycin, a broad-spectrum antibiotic notably effective against methicillin-resistant *Staphylococcus aureus* strains, represents the most significant drug class in drug-induced LABD and is responsible for over 50% of LABD cases (42). Amoxicillin and Clavulanate, widely used antibiotics, have been implicated in multiple cases of LABD (43–47). LABD seems more related to Amoxicillin than Clavulanate, as Clavulanate combined with ticarcillin has not been associated with LABD. However, the broad use of Amoxicillin and Clavulanate poses a challenge. Fewer cases of *β*-lactam-induced LABD prevent a definitive conclusion about the sole effect of Amoxicillin (47). Cases of LABD induced by Piperacillin and Tazobactam have also been frequently reported (19, 36, 48, 49). Antihypertensive drugs have also been identified as potential inducing agents (50). Amlodipine-induced LABD was first reported by Low et al. (51), with 56 cases reported in the FAERS database. Therefore, clinicians should be vigilant for potential AEs, including LABD, when administering Amlodipine to treat hypertension and other conditions, especially in elderly patients. A case of captopril-induced LABD was reported by Friedman et al. (52), while another case remained indeterminate due to the presence of concomitant medications (53). Captopril has ROR and PRR values of 99.12 and 98.87, supported by five case reports in the FAERS database. Bisoprolol, with a ROR of 6.92 and eight reports, has no supporting literature for the induction of LABD. Candesartan and Eprosartan have been linked to LABD (54), yet the FAERS database lacks any corresponding case reports. Some drugs, despite few reports, show high ROR and PRR values, suggesting an increased risk of LABD in specific patient populations. For example, Ampicillin and Sulbactam have ROR and PRR values of 137.77 and 137.28, with eight reports. This indicates that even with a relatively low number of reports, the association between certain drugs and LABD remains a concern. There are reports on three Ampicillin and Sulbactam cases (55–57) and one Captopril case. Drugs used to treat coronary heart disease, hypertension, hypercholesterolemia, such as Amlodipine (ROR 11.49) and Atorvastatin (ROR 6.7), although their ROR values are not as high as Vancomycin, require a balance between therapeutic efficacy and potential AEs. Additionally, there have been reports of LABD induced by immune checkpoint inhibitors such as anti-PD1 and anti-CTLA4 (58, 59), yet the FAERS database does not contain any corresponding reports for these medications. Our findings emphasize the importance of identifying and monitoring potential risk factors for LABD, especially with drugs known to increase LABD risk. Additionally, this underscores the importance of enhanced pharmacovigilance and clinical practice in detecting and assessing AEs of drugs. These data may also inform drug development and safety regulation, suggesting further research into mechanisms of drugs associated with increased risk of LABD and strategies to prevent or mitigate LABD. Lastly, our study suggests monitoring of drug AEs should occur throughout drug development and clinical trials, not just post-marketing, to identify and mitigate potential risks early.

This study leveraged the FAERS database to retrospectively assess the reported associations between drugs and LABD using pharmacovigilance methods. To our knowledge, this is the first real-world study utilizing the FAERS database to investigate drug-induced LABD. Our findings indicate significant correlations between LABD and various drug classes, including antibiotics, antifungal agents, analgesics, NSAIDs, cardiovascular medications, and calcium channel blockers. The results underscore the necessity for close patient monitoring when these drugs are clinically prescribed to promptly detect and manage potential AEs such as LABD. This research also sets the stage for future studies to explore the specific mechanisms of drug-induced LABD and to develop strategies for prevention and management of these adverse reactions (Table 4). It is important to note that this study has certain limitations. Firstly, the present study is retrospective in nature, thereby precluding the direct inference of a causal relationship between the medication and LABD from the outcomes. Secondly, there is a potential for reporting bias in the data, as AEs are reported voluntarily, which may lead to underreporting or overreporting, significantly influencing the ROR analysis. Thirdly, the reported association between the medication and LABD is confounded by comorbidities and concomitant medications. The data analysis included only the primary suspect medications. Fourthly, the FAERS database lacks comprehensive patient-level data, which limits the ability to assess confounding factors and conduct reliable statistical analyses. Lastly, the FAERS database does not provide detailed information on the timing of drug exposure, thus precluding the calculation of incidence rates and the quantification of individual effects of multiple drug exposures. Therefore, future research should consider prospective designs and more comprehensive data collection and analysis on drug exposure and the occurrence of LABD to further validate and expand upon our findings. In summary, this study provides crucial insights into the relationship between drugs and LABD and offers valuable guidance for drug utilization and patient management in clinical practice.

**Table 4:** Medications included in the present study and their mechanisms of action.

Medications investigated	Mechanism of action
Antibiotics	
Beta-lactams	
Ampicillin and Sulbactam	Cell wall inhibitor
Piperacillin and Tazobactam	Beta-lactamase inhibitor
Amoxicillin and Clavulanic acid	Cell wall synthesis blocker
Meropenem	Broad-spectrum inhibitor
Imipenem and Cilastatin	Cell wall synthesis disruptor
Cefuroxime	Peptidoglycan synthesis inhibitor
Ceftriaxone	Cell wall synthesis antagonist
Amoxicillin	Cell wall inhibitor
Glycopeptides	
Vancomycin	Peptidoglycan synthesis inhibitor
Macrolides	
Azithromycin	Ribosomal RNA blocker
Fluoroquinolones	
Ciprofloxacin	DNA replication inhibitor
Levofloxacin	Gyrase inhibitor
Sulfonamides	
Sulfamethoxazole and Trimethoprim	Folate synthesis inhibitor
Rifamycins	
Rifampin	Transcription inhibitor
Antifungals	
Terbinafine	Squalene epoxidase inhibitor
Fluconazole	Ergosterol synthesis inhibitor
Analgesics and NSAIDs	
Non-steroidal Anti-inflammatory Drugs (NSAIDs)	
Ketoprofen	COX enzyme inhibitor
Meloxicam	Selective COX inhibitor
Diclofenac	Prostaglandin synthesis blocker
Ibuprofen	Non-selective COX inhibitor
Analgesics/Antipyretics	
Paracetamol	Central COX inhibitor
Cardiovascular drugs	
ACE Inhibitors	
Captopril	Angiotensin II blocker
Calcium channel blockers	
Amlodipine	Vascular smooth muscle relaxant
Verapamil	Calcium channel blocker
Beta Blockers	
Bisoprolol	Adrenergic receptor antagonist
Diuretics	
Furosemide	Sodium reabsorption inhibitor
Statins	
Atorvastatin	Cholesterol synthesis inhibitor
Simvastatin	HMG-CoA reductase inhibitor
Antineoplastic drugs	
Gemcitabine	Nucleoside analog
Antiprotozoals	
Metronidazole	DNA strand breaker
Gout treatments	
Allopurinol	Uric acid production inhibitor
Antiepileptics	
Phenytoin	Sodium channel blocker
Cognitive enhancers	
Donepezil	Acetylcholine breakdown inhibitor
Proton pump inhibitors (PPIs)	
Omeprazole	Gastric acid secretion inhibitor

## Data Availability

The original contributions presented in the study are included in the article/supplementary material, further inquiries can be directed to the corresponding author.
